# Do spontaneous and mechanical breathing have similar effects on average transpulmonary and alveolar pressure? A clinical crossover study

**DOI:** 10.1186/s13054-016-1290-9

**Published:** 2016-04-28

**Authors:** Giacomo Bellani, Giacomo Grasselli, Maddalena Teggia-Droghi, Tommaso Mauri, Andrea Coppadoro, Laurent Brochard, Antonio Pesenti

**Affiliations:** Department of Health Science, University of Milan-Bicocca, Via Cadore, 48 20900 Monza, Italy; Department of Emergency and Intensive Care, San Gerardo Hospital, Monza, Italy; Department of Anesthesia, Critical Care and Emergency, Fondazione IRCCS Ca’ Granda - Ospedale Maggiore Policlinico, Milan, Italy; Department of Emergency and Intensive Care, A. Manzoni Hospital, Lecco, Italy; Keenan Research Centre, St. Michael’s Hospital, Toronto, ON Canada; Interdepartmental Division of Critical Care Medicine, University of Toronto, Toronto, ON Canada

**Keywords:** Mechanical ventilation, Transpulmonary pressure, Pressure support ventilation, Controlled ventilation, Esophageal pressure

## Abstract

**Background:**

Preservation of spontaneous breathing (SB) is sometimes debated because it has potentially both negative and positive effects on lung injury in comparison with fully controlled mechanical ventilation (CMV). We wanted (1) to verify in mechanically ventilated patients if the change in transpulmonary pressure was similar between pressure support ventilation (PSV) and CMV for a similar tidal volume, (2) to estimate the influence of SB on alveolar pressure (Palv), and (3) to determine whether a reliable plateau pressure could be measured during pressure support ventilation (PSV).

**Methods:**

We studied ten patients equipped with esophageal catheters undergoing three levels of PSV followed by a phase of CMV. For each condition, we calculated the maximal and mean transpulmonary (ΔP_L_) swings and Palv.

**Results:**

Overall, ΔP_L_ was similar between CMV and PSV, but only loosely correlated. The differences in ΔP_L_ between CMV and PSV were explained largely by different inspiratory flows, indicating that the resistive pressure drop caused this difference. By contrast, the Palv profile was very different between CMV and SB; SB led to progressively more negative Palv during inspiration, and Palv became lower than the set positive end-expiratory pressure in nine of ten patients at low PSV. Finally, inspiratory occlusion holds performed during PSV led to plateau and Δ P_L_ pressures comparable with those measured during CMV.

**Conclusions:**

Under similar conditions of flow and volume, transpulmonary pressure change is similar between CMV and PSV. SB during mechanical ventilation can cause remarkably negative swings in Palv, a mechanism by which SB might potentially induce lung injury.

**Electronic supplementary material:**

The online version of this article (doi:10.1186/s13054-016-1290-9) contains supplementary material, which is available to authorized users.

## Background

In recent years, conflicting data have been published concerning the beneficial or detrimental effect of preserved spontaneous breathing (SB) compared with fully controlled mechanical ventilation (CMV) during acute respiratory failure [1–5]. SB has been credited with having several beneficial effects, such as improved hemodynamics [6], improved ventilation-to-perfusion matching [7], decreased ventilator-induced lung injury (VILI) [8, 9], and decreased muscle atrophy [10]. However, SB can also cause or aggravate lung injury during mechanical ventilation, as shown by experimental evidence [11–13], by mechanisms that include negative intrathoracic and alveolar pressure (causing interstitial or alveolar edema), loss of control over tidal volume (V_T_), and inhomogeneous regional stretch. Abolition of SB might be one of the mechanisms by which the use of neuromuscular blocking agents in the first hours after intubation may improve patient outcome [14]. For this reason, greater attention is now being paid to better understanding of the pressure across the lung (i.e., transpulmonary pressure [P_L_]).

While airway pressure (Paw) is usually lower during SB than during CMV, this does not necessarily translate into a lower pressure across the lung (i.e., a lower P_L_). By convention, P_L_ is the difference between the pressure at the airway opening and the pleural or esophageal pressure [15]. Our hypothesis was that, for a given inspired volume and flow, and for the same mechanical properties (i.e., compliance and resistance) of the lung, the amplitude of the change in P_L_ (ΔP_L_) during assisted SB and during CMV should not differ, regardless of the level of inspiratory effort, whereas the absolute value of airway and esophageal pressure should differ. To our knowledge, however, despite the major implication of understanding the mechanisms of VILI, this has not been directly verified in patients. For instance, the net effect on local transmural vascular pressures may significantly impact the generation of VILI [16]. The total P_L_ can be divided into the pressure generated to overcome the resistance to airflow between the airway opening and the alveoli, and the pressure needed to expand the terminal airways (i.e., the transalveolar pressure). Only the latter part of the P_L_, which equals the product of lung elastance and volume, is dissipated across the alveolus and is commonly considered to cause VILI [17]. At the same time, the pathophysiological relevance of the pressure needed to generate the airflow across the airway will be substantially different between CMV and assisted SB, which may have clinical consequences. The absolute value of the pressure surrounding the lungs, as well as that of the alveolar pressure (Palv), will change in a positive direction related to atmosphere during controlled ventilation and in a more negative direction for increasing levels of breathing effort. Airflow generation in the presence of elevated airflow resistance may lead to an extremely high P_L_ during both fully controlled and spontaneously assisted ventilation, but accompanied in the latter case by very negative pressure around and even inside the alveoli.

The purpose of the present study was to compare the ΔP_L_ during spontaneous assisted breathing and fully controlled ventilation, trying to match similar conditions of airflow and volume, in a group of patients undergoing different levels of pressure support ventilation (PSV) followed by a phase of CMV. Moreover, we investigated the role of the resistive pressure in the generation of negative intrathoracic and intraalveolar pressure during spontaneous assisted breathing. Finally, we reasoned that if the transalveolar pressure is similar during CMV and PSV, then plateau pressure (Pplat) during PSV, obtained in the absence of flow and during patient’s muscle relaxation, should provide similar information as during CMV. For this reason, we evaluated the reliability of the measurement of Pplat during PSV compared with that obtained during CMV.

## Methods

This is a secondary analysis of data collected in a previous study, where the methods are described in more detail [18].

### Patients

The protocol was approved by our institution’s ethics committee (San Gerardo Hospital, Monza, Italy). We enrolled patients admitted to our general intensive care unit who were orotracheally intubated and undergoing PSV or neurally adjusted ventilatory assist with a positive end-expiratory pressure (PEEP) level greater than 5 cmH_2_O. Exclusion criteria were age less than 18 years, hypoxemia requiring a fraction of inspired oxygen (FiO_2_) greater than 60 %, presence of bronchopleural air leaks, hemodynamic instability requiring vasopressors, Richmond Agitation and Sedation Scale score less than −1, history of chronic obstructive pulmonary disease, and clinical suspicion or evidence of intrinsic PEEP. Informed consent to participate in the study was obtained (immediately or in delay, according to the institutional ethics committee’s recommendations) from all patients.

### Study phases

After patients were enrolled, we inserted a nasogastric tube with electrodes and an esophageal balloon. The correct placement of the esophageal balloon was verified according to the standard procedure [15]. The waveforms of Paw, esophageal pressure (Pes), airflow, and electrical activity of the diaphragm were continuously recorded using a data acquisition system (PowerLab; AD Instruments, Colorado Springs, CO, USA) at a sampling frequency of 100 Hz for offline data analysis.

The study protocol consisted of three consecutive phases. Initially, patients underwent a trial of PSV at three levels of assistance averaging 13.6 ± 3.1 cmH_2_O, 7.6 ± 3.1 cmH_2_O, and 2.4 ± 2.1 cmH_2_O (hereinafter defined as high, medium, and low, respectively) separated by intervals of 4 cmH_2_O, as previously described [19], lasting 30 minutes each in random order. During each level of PSV, at 10-minute intervals, we performed two short (2–3 seconds) end-expiratory and end-inspiratory occlusions, verifying adequate relaxation of the muscles by a return of the electrical activity of the diaphragm signal at baseline and a flat, stable plateau in the Paw waveform. After a phase of neurally adjusted ventilatory assist ventilation (not taken into account for the present analysis), patients were sedated and switched to CMV with a V_T_ of 6–8 ml/kg and two different constant (square waveform) flow rates of 0.5 and 1 L/second. In this phase, we carefully verified the absence of SB activity by observing esophageal and electrical activity waveforms. Also in this phase, three end-expiratory and end-inspiratory occlusions were performed. Throughout the study protocol, PEEP and FiO_2_ were left unchanged from the clinically set values.

### Data analysis

#### Signals measured and calculated

**Fig. 1:**
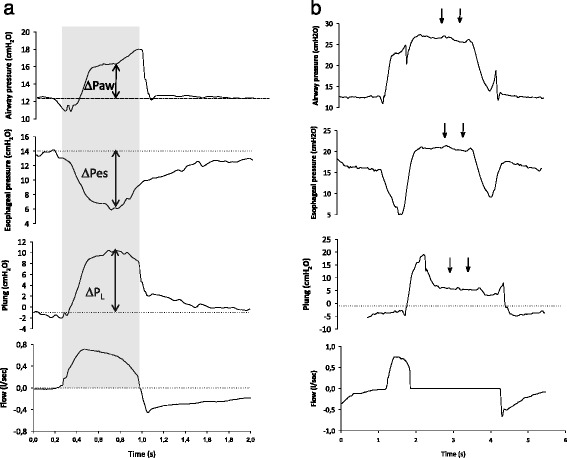
Individual examples of airway and esophageal pressure tracings of pressure support breaths sampled during regular tidal ventilation (**a**) and one prolonged inspiratory hold (**b**). **a** For each selected breath, from the airway (ΔPaw) and esophageal (ΔPes) pressure swings we calculated the transpulmonary lung pressure (Plung) swings (ΔP_L_) as changes from the end expiration (*dotted lines*) at two time points of interest: the point of maximum ΔP_L_ and the mean over inspiration (*gray rectangular area*). **b** Following an inspiratory hold, when the patient relaxes the inspiratory muscles, a plateau is seen in airway and esophageal pressure (*arrows*), whose differences from the end-expiratory level represent the elastic recoil pressure of the respiratory system and of the chest wall, respectively

For the present study, the electrical activity of the diaphragm data and the periods of neurally adjusted ventilatory assist ventilation were not taken into account. Continuous tracings of P_L_ were generated as Paw – Pes, where Paw and Pes are airway and esophageal pressure, respectively. As we preferred to avoid using absolute values for Pes, we always refer to ΔP_L_ from the end-expiratory level (Fig. 1a). Analogously, a Pes decrease or increase (ΔPes) was calculated as the difference in Pes from the end-expiratory level (Fig. 1a).

The pressure generated by inspiratory muscles (Pmus) was calculated as the difference between the Pes and the theoretical curve of elastic recoil of the chest wall, which was in turn calculated by instant-by-instant multiplication of the volume signal by the chest wall elastance obtained during the phase of CMV. Palv was calculated as Paw − (flow/Raw), where Raw is the airway resistance measured during CMV and its changes (ΔPalv) were calculated as the difference between end expiration and the value of Palv at the selected time points. Finally, the changes in transalveolar pressure due to the elastic recoil pressure of the lung (ΔP_L,el_) were calculated as ΔPalv − ΔPes at the selected time points. At the end of inspiration, when the inspiratory flow is instantaneously zero, the transpulmonary and transalveolar pressures are equal [15]. If the V_T_ is kept constant during spontaneous and controlled ventilation, the pressure across the alveolus must be the same.

#### Time points sampled and averaging of breaths

For regular V_T_, we sampled ten inspirations for the phase of CMV and ten inspirations for each level of PSV (attempting to identify ten breaths with a V_T_ of a size as close as possible to those used during CMV), avoiding those parts of the tracings with a low quality of the Pes signal (e.g., peristaltic waves).

For each breath, we sampled two conditions (Fig. 1a):

1. The time point at which ΔP_L_ was maximum (maximum-ΔP_L_)
2. The mean value over inspiration (mean-insp)

The values from the ten breaths sampled during each condition were then averaged, obtaining in each patient four values (CMV, PSV-high, PSV-medium, and PSV-low) in two conditions each (maximum-ΔP_L_ and mean-insp), which were later used for analysis. After matching for the V_T_ size, we attempted to match inspirations obtained in PSV with those with similar mean inspiratory flows in CMV. Since the matching for inspiratory flow was rather poor (see below) due to the availability of only two inspiratory flows in CMV (0.5 and 1 L/second), we performed an analysis focused only on combinations of similar (arbitrarily defined as an absolute difference <0.1 L/minute) mean inspiratory flows between PSV and CMV.

#### Measurement of plateau pressure

For those breaths in which an inspiratory hold was obtained, we measured plateau Paw and Pes, and static respiratory system compliance was calculated according to standard formulas during both CMV and PSV. During PSV offline analysis, we paid particular attention to avoiding those occlusions in which a clean plateau could not be identified in the Paw profile (Fig. 1b). To define a “clean” plateau, we discarded those measures in which the Paw tracing was not flat (i.e., steadily increasing or decreasing or with periodic oscillations greater than 2–3 cmH_2_O).

### Statistical analysis

Data are expressed as mean ± standard deviation unless specified otherwise. Analyses were performed using SPSS software (IBM, Armonk, NY, USA). Comparisons between the four study phases (CMV and the three levels of support) were performed using one-way analysis of variance (ANOVA) for repeated measures. Correlation between variables was assessed by linear regression. Mean bias and 95 % confidence intervals for agreement between measurement obtained during CMV and PSV of plateau airway pressure and ΔP_L,el_ were calculated according to the method of Bland and Altman. *p* < 0.05 was considered statistically significant.

## Results

The main demographic and clinical variables of the patients are reported in Table 1. All patients completed the protocol and had data available for all study phases, except for 9.5 % of the inspiratory holds, which were discarded (see below). Inspiratory flows and V_T_ did not significantly differ overall during the three levels of PSV and the CMV phases (Table 2), with a slight decrease during PSV-low. V_T_ in PSV and CMV were tightly correlated (*r*^2^ = 0.93, data not shown), but the correlation was loose for inspiratory flow (*r*^2^ = 0.23, data not shown), suggesting that, while on average there were no differences between PSV and CMV at an individual breath level, the conditions might not be comparable.

**Table 1:** Patient characteristics

Characteristic	Data
Age, years	66 ± 14
Male sex, %	50 %
ICU survival, %	60 %
Days since intubation	5 ± 4
Compliance, ml/cmH_2_O	43 ± 12
PaO_2_/FiO_2_, mmHg	224 ± 59
PaCO_2_, mmHg	44.6 ± 6.3
SAPS II score	43 ± 12

*FiO*_*2*_ fraction of inspired oxygen, *ICU* intensive care unit, *PaCO*_*2*_ partial pressure of carbon dioxide, *PaO*_*2*_ partial pressure of oxygen, *SAPS II* Simplified Acute Physiology Score II

Data are expressed as mean ± standard deviation unless specified otherwise

**Table 2:** Average tidal volume, inspiratory flow, and muscle pressure during the study phases

	Level of assistance					
	CMV	PSV-high	PSV-medium	PSV-low	Overall	*p* Value^a^
Tidal volume, ml	475 ± 121	479 ± 119	461.9 ± 125	449 ± 126	466 ± 118	0.95
Flow, L/min	0.49 ± 0.1	0.47 ± 0.13	0.47 ± 0.12	0.46 ± 0.16	0.47 ± 0.13	0.94
Pmus, cmH_2_O	0.34 ± 0.48	2.75 ± 1.41	5.42 ± 2.26	8.3 ± 2.59	4.2 ± 3.5	<0.001

*CMV* controlled mechanical ventilation, *PSV* pressure support ventilation, *Pmus* inspiratory muscle pressure

^a^ *p* values were calculated using repeated measures analysis of variance

### Comparison of transpulmonary and alveolar pressures during CMV and PSV

**Fig. 2:**
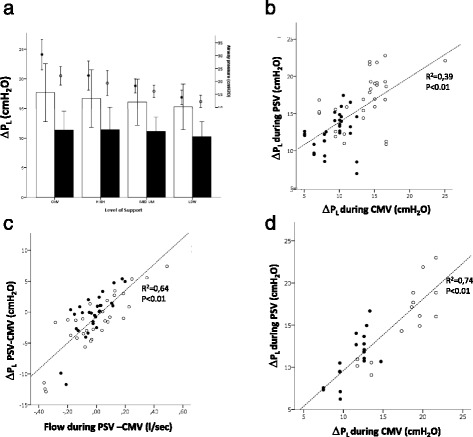
**a** Pressures (transpulmonary pressure swings [ΔP_L_] on the left, shown as *bars* and standard deviation), versus the level of support (high, medium, or low). It shows that, by contrast with airway pressure, ΔP_L_ swings during inspiration were similar and not statistically different between controlled mechanical ventilation (CMV) and any of the support levels. *Closed symbols* represent mean values during inspiration, and *open symbols* show maximum values during inspiration. The correlation between the value recorded during CMV and pressure support ventilation (PSV), albeit significant, was poor (**b**). The difference between the measurement of ΔP_L_ obtained during CMV and PSV was explained largely by the corresponding difference in the inspiratory flow (**c**), as shown by the highly significant correlation. Indeed, when the analysis was restricted to the breaths with similar inspiratory flows (i.e., with an absolute difference less than 0.1 L/minute) (**d**), the correlation of ΔP_L_ obtained during CMV and PSV became very tight

As expected (Fig. 2a), switching from CMV to decreasing levels of PSV led to a significant reduction in airway pressure (*p* < 0.001 by ANOVA). Simultaneously, Pmus was almost zero during CMV, and application of decreasing levels of PSV led to a progressive increase of Pmus (*p* < 0.001 by ANOVA) (Table 2).

**Fig. 3:**
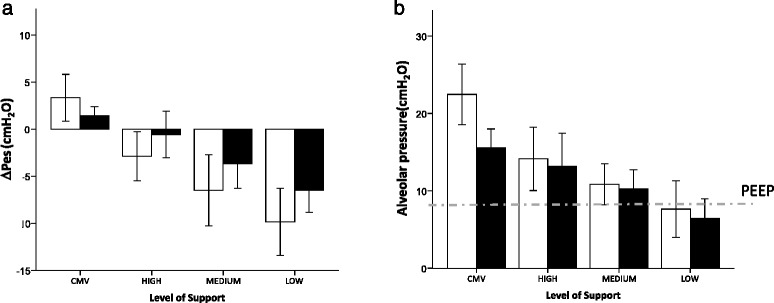
The swings of esophageal pressure (ΔPes) from baseline (**a**), shown as mean and standard deviation, were positive during controlled mechanical ventilation (CMV) but became negative during pressure support ventilation and progressively lower for decreasing levels of support (high, medium, and low). This was the case when we considered both the mean values during inspiration (*closed bars*) and at the moment of maximum transpulmonary pressure (*open bars*). Similarly, alveolar pressure (Palv) (**b**), shown as bars and standard deviation, progressively decreased from CMV through the different levels of pressure support ventilation (high, medium, and low). Moreover, Palv was, on average, lower than the set positive end-expiratory pressure (PEEP) (*dashed line*), both as a mean during inspiration (*closed bars*) and at the moment of maximum transpulmonary pressure (*open bars*), if a low level of support was applied

As a result, both mean and maximal ΔP_L_ values were similar overall between CMV and all the levels of PSV applied, as shown in Fig. 2a (*p* = n.s. for both by ANOVA). The individual linear correlation between ΔP_L_ measured during PSV and CMV was, however, quite poor (Fig. 2b), indicating a wide dispersion (Fig. 2b). The individual differences between measurements of ΔP_L_ were well explained by the differences in flow rates between PSV and CMV breaths, as shown in Fig. 2c, indicating that the resistive pressure drop caused this difference. We thus repeated the analysis, focusing only on values (either maximum-ΔP_L_ or mean-insp) for which the inspiratory flow was similar, defined as having an absolute difference less than 0.1 L/minute. This resulted in a tight correlation, very close to the line of identity between the ΔP_L_ values measured during CMV and PSV (Fig. 2d). ΔPes was positive during CMV, whereas decreasing levels of PSV assistance led to progressively more negative inspiratory ΔPes, as shown in Fig. 3a (*p* < 0.01 for both time points by ANOVA).

**Fig. 4:**
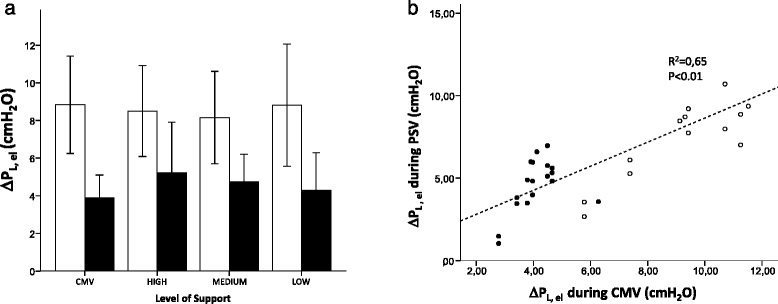
The transalveolar pressure (i.e. the pressure distending the alveoli), which is caused by the elastic recoil of the lungs (ΔP_L,el_), was similar and nonsignificantly different between controlled mechanical ventilation (CMV) and any of the support levels of pressure support ventilation (high, medium, and low), either as a mean value (*open symbols*) or as a maximum value (*closed symbols*), during inspiration (**a**), shown as mean and standard deviation. The values measured during pressure support ventilation (PSV) and CMV were very closely correlated and close to the line of identity (*dashed line*), as shown in (**b**) (analysis restricted to breaths with similar inspiratory flow)

Our findings were similar for Palv (Fig. 3b) (*p* < 0.01 for both time points by ANOVA), which became progressively lower for decreasing levels of assistance. In particular, we found a mean-insp Palv that was below the set PEEP in nine of ten patients during PSV-low, in two of ten during PSV-medium, and in one of ten during PSV-high. Transalveolar pressure (which equals ΔP_L,el_) was not different between CMV and any condition of PSV (Fig. 4a) (*p* = n.s. for both by ANOVA). Moreover, similarly to ΔP_L_, we found a tight correlation between ΔP_L,el_ measured during CMV and during PSV (Fig. 4b).

### Value of plateau pressure measured during PSV

**Fig. 5:**
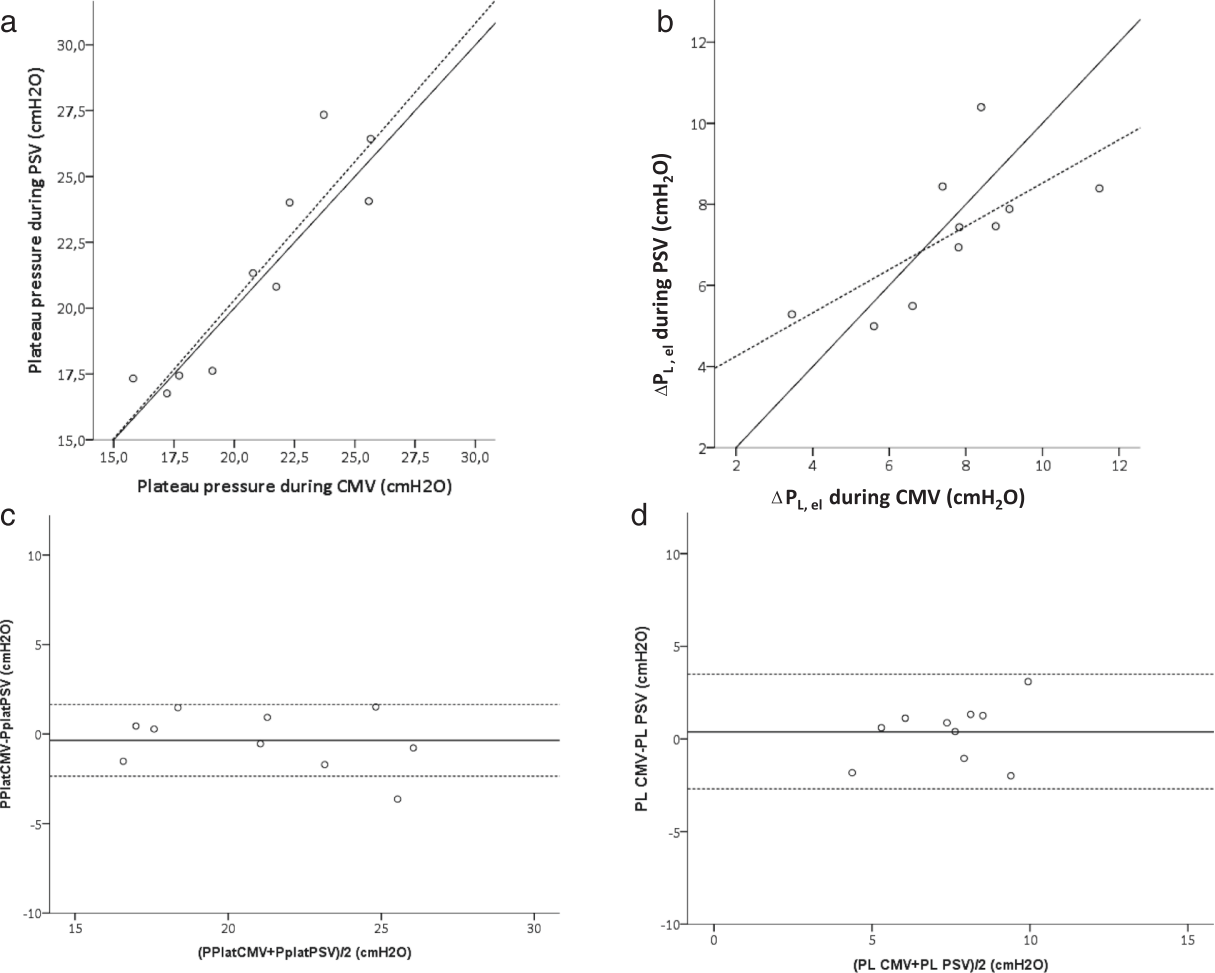
Tight correlation (*solid line*, very close to the identity, represented by the *dashed line*) was found between the values obtained during an inspiratory hold obtained while under controlled mechanical ventilation (CMV) and pressure support ventilation (PSV) for plateau airway pressure (Pplat) in the airways (**a**), static transpulmonary pressure (ΔP_L,el_) (**b**), and the respective Bland-Altman analyses (**c** and **d**), showing mean bias (*solid line*) and 95 % confidence intervals (*dotted lines*)

To estimate the pressure effectively distending the respiratory system and the lungs during PSV, we performed prolonged (about 2 seconds) inspiratory holds, aiming to obtain a period of zero Pmus, hence similar to CMV. Both plateau airway pressure and ΔP_L,el_ measured during inspiratory holds performed after PSV breaths were highly correlated with the value measured in the same patient during CMV, as shown in Fig. 5, albeit a greater scatter was present for ΔP_L_. Mean differences and 95 % CIs were 0.35 (−2.8 to 3.5) cmH_2_O for plateau airway pressure and 0.38 (−2.7 to 3.5) cmH_2_O for ΔP_L,el_ (Fig. 5). Similar results were found for compliance. The measurements obtained during PSV were tightly correlated with those obtained in the same patient during CMV with mean differences and 95 % CI of 3.3 (−17 to 11) ml/cmH_2_O for respiratory system compliance and −2 (−59 to 56) ml/cmH_2_O for lung compliance. In this case, the values were very close to the identity line for values of lung compliance up to 100 ml/cmH_2_O (plots are shown in figure E1 Additional file 1).

## Discussion

Our data show that, in a mixed population of patients undergoing spontaneous assisted breathing, neither transpulmonary nor transalveolar pressure changes differed between controlled and spontaneous assisted ventilation for comparable volumes and flows. When the breaths were matched for inspired volume and inspiratory flow, the values were almost identical. This was also the case in static conditions, suggesting that Pplat can be measured reliably also during PSV. By contrast, the absolute Palv value could markedly differ during inspiration, frequently becoming lower than PEEP during PSV.

A first consequence is that if SB has any role (protective or detrimental) in modulating VILI, this cannot be mediated by a pure, isolated difference in the ΔP_L_. Moreover, it has to be considered that only part of the P_L_ is dissipated across the alveoli; this is called the transalveolar pressure and equals the product of lung elastance and volume. The remaining pressure determines a gradient between the airway opening and the alveoli, which yields an inspiratory flow. This gradient will be the same, regardless of the amount of pressure generated by the patient and by the ventilator, as shown by our data (Fig. 3), and it depends on the airflow profile and inspiratory Raw. For example, Yoshida et al. defined the “total alveolar stretching pressure” (P_L_) as Pplat + ΔPes (ΔPes being measured during inspiration), but this pressure extends across the entire lung (alveoli + airways) and is not specific for “alveoli” [11]. As a matter of fact, the increased P_L_ in the presence of a strong inspiratory effort is associated with an increased resistive pressure [11].

These concepts are illustrated in the figure E2 in Additional file 1, which shows the respective meaning of transpulmonary and transalveolar pressure under assisted SB and CMV. For the same mechanical properties (compliance and resistance) of the respiratory system, the ΔP_L_ will differ between controlled and SB only if flow and/or lung volume differ, and transalveolar pressure will differ only if lung volume changes. As shown by our data, in the presence of similar volumes and inspiratory flows, transpulmonary and transalveolar pressure do not differ between controlled and spontaneous ventilation, regardless of the inspiratory effort.

However, there are strong data in the literature showing that, under some conditions, SB efforts can be detrimental. In keeping with our findings, this can be explained by at least three mechanisms that do not imply a different transpulmonary or transalveolar pressure change. First, in all the pressure-targeted ventilatory modes, the control over V_T_ is lost and, consequently, the patient might develop nonprotective V_T_ even if airway pressures are not high. This is possibly one of the mechanisms explaining the report of Bruells et al. [20], who found a more severe degree of VILI if negative pressure ventilation (similar in some aspects to SB) was used, as compared with positive pressure ventilation. Since in this elegant experiment the V_T_ was not controlled, it was probably higher in the negative pressure ventilation group, as suggested by the lower arterial PaCO_2_. In such circumstances, the ΔP_L_ will be higher during negative pressure (spontaneous) ventilation, albeit, as emphasized before, this is simply due to the higher V_T_ reached [15].

Second, absolute values of esophageal, pleural, alveolar, and intrathoracic pressure will be progressively lower during strenuous breathing efforts, leading, in some cases, to values below PEEP for the entire respiratory cycle, as shown by our data. The consequences of these negative pressure swings can be profound, particularly regarding the hemodynamic profile. In fact, while during CMV the resistive pressure drop does not have major physiological consequences, during assisted SB the inspiratory resistive pressure drop (unlikely to be compensated by the expiratory pressure drop of opposite sign, usually passive and driven mainly just by the elastic pressure recoil of the chest wall) causes major physiological consequences. It increases the filling of the right heart, impairs the function of the left ventricle, and causes a negative interstitial pressure in the lung, which can in turn lead to fluid accumulation in the pulmonary interstitium. Moreover, the increased cardiac output usually associated with SB [6] will necessarily lead to an increased perfusion of lung capillaries, the latter being a known factor contributing to VILI [21], even in the absence of increased vascular pressures [22]. During inspiration, the fall in pleural pressure is larger than the fall in intravascular pressure in the pulmonary circulation, explaining the increase in transmural pressure increases [23]. This increase in vascular pressure in the pulmonary circulation has been shown to favor the development of VILI [16].

Toumpanakis and coworkers [24] imposed a resistive load on spontaneously breathing animals, causing important negative inspiratory pressure swings, and they found severe lung injury. It is worth noting that, in this model, even if the V_T_ were not measured, this was likely normal; thus, the transalveolar pressure (product of inspired volume and elastance) was normal, but with very negative absolute alveolar and intrathoracic pressures. Similarly, Stalcup and Mellins previously demonstrated that, during asthma, negative pleural pressure swings cause alveolar fluid accumulation [25].

Finally, the pleural pressure during SB might be uneven due to the action of diaphragmatic contraction, leading to “regional” overinflation and or pendelluft, as recently shown by electrical impedance tomography [12].

In this study, we focused on PSV, a ventilatory form that (except during asynchronies) implies a relative stereotyped interaction between patient and ventilator: The ventilator delivers flow simultaneously with the patient’s demand. However, patient-ventilator interaction can be more complex during other ventilatory modes allowing SB, such as synchronized intermittent mandatory ventilation, bilevel ventilation, and airway pressure release ventilation [26]. In these conditions, P_L_ and Palv changes can be greatly amplified. As an example, while breath stacking [27] will lead to increased P_L_, an inspiratory effort occurring during expiration will cause a profound negative Palv.

This study has some limitations. First, it was originally designed not to specifically test this hypothesis but to evaluate the relationship between diaphragmatic electromyogram and muscle pressure. However, the data collected (Pes and Paw during controlled and spontaneously assisted ventilation) are reliable and allowed us to design this independent study. The sample size was relatively small (ten patients) and had some heterogeneity. Thanks to the crossover study design, we calculated (based on the standard deviation of P_L_ during CMV) that the minimum detectable difference of P_L_ between two steps was 3.6 cmH_2_O, with an α of 0.05 (two-tailed) and a power of 80 %.

A second limitation is that our calculations were based on a single value of Pes and on the assumption of a single compartment model. This is a simplification, since it is known that, particularly in the presence of lung disease, pleural pressure is not uniform and parenchymal compliance and resistance can have regional heterogeneities. As a consequence, our results should be regarded as “average” values for the lungs, but we have to keep in mind that, for some lung regions, P_L_ (or transalveolar) pressures can be considerably higher (or lower). The same reasoning also applies to the end-inspiratory occluded pressure during pressure support, which represents an average of the pressures distending the alveoli.

A third limitation resides in the fact that we applied two fixed squared flow rates in CMV without a prospective match with PSV for airflow value and shape. Consequently, while V_T_ was very similar during the two ventilation modes, airflow was not, without a systematic direction. To overcome this limitation in part, we performed ex post facto matching by focusing part of the analysis on the breaths with similar airflow values. Moreover, also based on previous data from our group [28], we did not expect that Raw would present relevant differences between these two conditions, and we assumed them to be identical.

Finally, we assumed that compliance and resistance of the respiratory system did not change between CMV and PSV, but different volume history or the use of sedation (which was difficult to avoid, however) might have affected respiratory mechanics.

## Conclusions

We show that ΔP_L_ under similar conditions of inspiratory flow and volume is similar between fully controlled and assisted mechanical ventilation. However, in the latter condition, the Pes and Palv can have remarkable negative swings that are in part required to overcome the airflow resistive pressure. Negative Palv values and their consequences on fluid shifts are potential mechanisms by which SB might induce lung injury. Finally, we show that by performing an inspiratory hold, it is possible to obtain a good estimate of the total pressure distending the respiratory system (or the lung) also during PSV.

## Key messages

- Transpulmonary and transalveolar pressures do not have the same meaning.
- Under similar conditions of flow and volume, spontaneous assisted breathing leads to transpulmonary pressures similar to those of controlled ventilation.
- During spontaneous breathing, the pressure drop due to airflow resistance leads to negative drops in alveolar pressure.
- The total elastic pressure distending the respiratory system can also be reliably measured during pressure support ventilation by means of an end-inspiratory hold.

## Additional file

Additional file 1: The electronic supplement contains figures E1 and E2. (DOCX 190 KB)

## Abbreviations

ANOVA: analysis of variance
CMV: controlled mechanical ventilation
FiO_2_: fraction of inspired oxygen
ICU: intensive care unit
PaCO_2_: partial pressure of carbon dioxide
Palv: alveolar pressure
PaO_2_: partial pressure of oxygen
Paw: positive airway pressure
PEEP: positive end-expiratory pressure
Pes: esophageal pressure
P_L_: transpulmonary pressure
P_L,el_: elastic recoil pressure of the lung
Pmus: pressure generated by inspiratory muscles
Pplat: plateau pressure
PSV: pressure support ventilation
Raw: airway resistance
RM-ANOVA: repeated measures analysis of variance
SAPS II: Simplified Acute Physiology Score II
SB: spontaneous breathing
VILI: ventilator-induced lung injury
V_T_: tidal volume
ΔPalv: change in alveolar pressure
ΔP_L_: change in transpulmonary pressure
