# Supplementary material for: Do spontaneous and mechanical breathing have similar effects on average transpulmonary and alveolar pressure? A clinical crossover study
Source: Crit Care. 2016 Apr 28;20:142. doi: 10.1186/s13054-016-1290-9 (PMC4862136; doi:10.1186/s13054-016-1290-9)
Supplement: Additional file 1: — The electronic supplement contains figures E1 and E2. (DOCX 190 KB) [file 13054_2016_1290_MOESM1_ESM.docx]

**Do spontaneous and mechanical breathing have similar effects on average transpulmonary and alveolar pressure? A clinical crossover study**

Giacomo Bellani MD PhD^1,2^, Giacomo Grasselli, MD^2^, Maddalena Teggia-Droghi, MD^1,2^, Tommaso Mauri, MD^3^, Andrea Coppadoro, MD^4^, Laurent Brochard, MD^5,6^, Antonio Pesenti, MD^1,2^

1. Department of Health Science, University of Milan-Bicocca, Monza, Italy;

2. Department of Emergency and Intensive Care, San Gerardo Hospital, Monza, Italy

3. Department of Anesthesia, Critical Care and Emergency, Fondazione IRCCS Ca’ Granda Ospedale Maggiore Policlinico, Milan, Italy

4. Department of Emergency and Intensive care, A. Manzoni Hospital, Lecco, Italy

5. Keenan Research Centre, St Michael’s Hospital, Toronto, Canada

6. Interdepartmental Division of Critical Care Medicine, University of Toronto, Toronto, Canada

**Electronic supplemental Material**

**
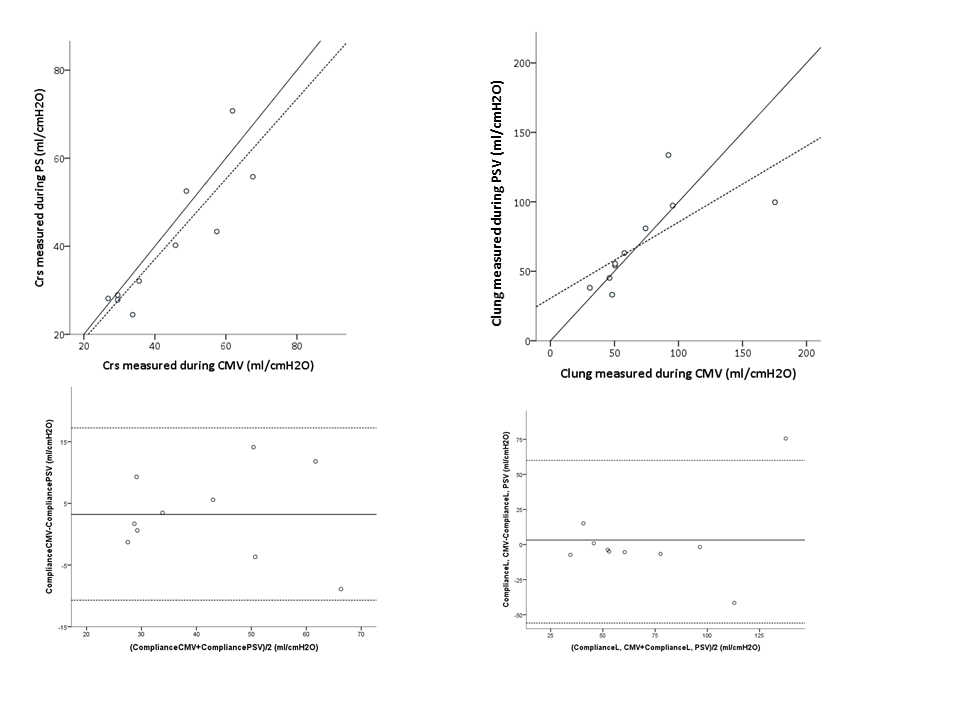
**

**Figure E1:** The figure shows the tight correlation between the values obtained during an inspiratory hold obtained while under controlled (CMV) and pressure support ventilation (PSV) for the compliance of the respiratory system (Panel A) and of the lung (Panel B), and the respective Bland and Altman analysis (panels C and D), for lung compliance measurements obtained in PSV and CMV were very similar for values <100 ml/cmH_2_O.

**
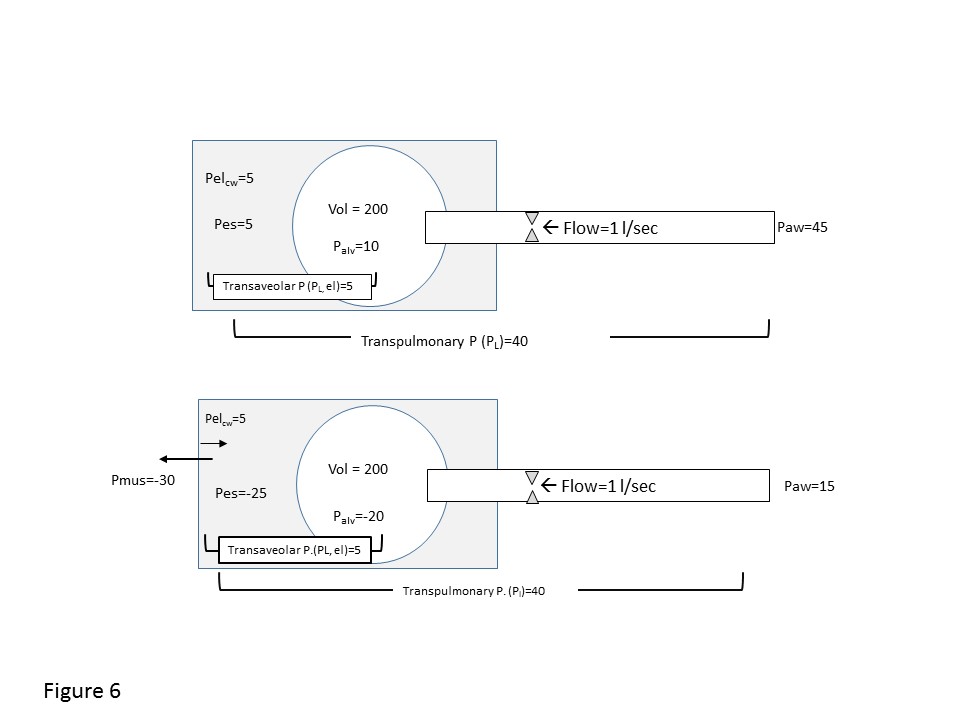
**

**Figure E2:** The figure shows the theoretical pressures for fully controlled mechanical ventilation (upper panel) and a pressure supported breath (lower panel), under the assumption of identical mechanical properties of the respiratory system. For the same inspiratory flow, the transpulmonary pressure (P_L_), which is the difference between airway pressure (Paw) and esophageal pressure (Pes), will be the same during CMV and PSV (35 cmH_2_O, in this example). Similarly, if the lung is at the same volume the pressure across the alveolar wall, which is due to the elastic recoil of the lung (P_L, el_=Palv-Pes), will not differ between CMV and PSV. However, with spontaneous breathing, the alveoli will be surrounded by a negative pressure (Pes), which equals the algebraic sum of the elastic recoil pressure of the chest wall (Pel, cw) and the pressure generated by the inspiratory muscles (Pmus). Moreover, during PSV, the alveolar pressure (Palv) will become very negative to overcome the resistive pressure drop.
